# Multiple Shades of Gray—Macrophages in Acute Allograft Rejection

**DOI:** 10.3390/ijms24098257

**Published:** 2023-05-04

**Authors:** Katharina Lackner, Susanne Ebner, Katrin Watschinger, Manuel Maglione

**Affiliations:** 1 Daniel Swarovski Research Laboratory, Department of Visceral, Transplant and Thoracic Surgery, Medical University of Innsbruck, 6020 Innsbruck, Austria; katharina.lackner@i-med.ac.at (K.L.); susanne.ebner@i-med.ac.at (S.E.); 2 Institute of Biological Chemistry, Biocenter, Medical University of Innsbruck, 6020 Innsbruck, Austria; katrin.watschinger@i-med.ac.at; 3 Department of Visceral, Transplant, and Thoracic Surgery, Medical University of Innsbruck, 6020 Innsbruck, Austria

**Keywords:** macrophages, polarization, acute rejection, immunosuppression, transplantation, tetrahydrobiopterin, alkylglycerol monooxygenase

## Abstract

Long-term results following solid organ transplantation do not mirror the excellent short-term results achieved in recent decades. It is therefore clear that current immunosuppressive maintenance protocols primarily addressing the adaptive immune system no longer meet the required clinical need. Identification of novel targets addressing this shortcoming is urgently needed. There is a growing interest in better understanding the role of the innate immune system in this context. In this review, we focus on macrophages, which are known to prominently infiltrate allografts and, during allograft rejection, to be involved in the surge of the adaptive immune response by expression of pro-inflammatory cytokines and direct cytotoxicity. However, this active participation is janus-faced and unspecific targeting of macrophages may not consider the different subtypes involved. Under this premise, we give an overview on macrophages, including their origins, plasticity, and important markers. We then briefly describe their role in acute allograft rejection, which ranges from sustaining injury to promoting tolerance, as well as the impact of maintenance immunosuppressants on macrophages. Finally, we discuss the observed immunosuppressive role of the vitamin-like compound tetrahydrobiopterin and the recent findings that suggest the innate immune system, particularly macrophages, as its target.

## 1. Introduction

Over the last five decades, organ transplantation has developed from an experimental treatment strategy to standard of care for end-stage failure of vital organs. Continuous advances in preservation strategies, surgical techniques, perioperative antibiotic prophylaxis and anesthesia were and are still pivotal for this development [1,2,3,4]. However, the major cornerstone for its success was the introduction of modern immunosuppression starting with cyclosporine A (CsA) in the 1980s [5,6]. Despite constant improvements, drug toxicities resulting from lifelong maintenance immunosuppressive medication still compromise graft and recipient survival, resulting in unvaried long-term attrition of transplanted organs that does not reflect the excellent short-term improvements [7]. Additionally, important adverse effects of lifelong immunosuppression such as hepatic and renal toxicity, hyperlipidemia and infections hamper graft and patient survival, limiting their quality of life [8,9,10,11]. As a result, currently available maintenance immunosuppressive protocols do not meet the required clinical needs and identifying novel molecular targets and pathways to improve long-term graft survival remains of utmost importance [12,13,14].

Historically, the research addressing allograft rejection mainly focused on the adaptive immune system, increasing the knowledge regarding different cell subsets involved in this process [15,16]. This goes back to early publications showing that T-cells played a crucial role in allograft rejection [17,18]. As a result, inhibiting differentiation and proliferation of the activated adaptive immune cells and their products became the focus of various immunosuppressive agents that entered the market [19].

Even though the presence of mononuclear cells infiltrating the rejected graft was already described in the 1980s [20] and macrophages were found to make up 60% of these infiltrating cells in organs undergoing acute rejection [21], cells of the innate immune system are only nowadays increasingly recognized as crucial contributors in transplant immunology [22]. Macrophages are among the first cells populating injured tissue, such as a transplanted organ [23]. Thanks to a myriad of diverse surface receptors such as pattern recognition receptors (PRR) that recognize damage-associated molecular patterns (DAMPs), macrophages locate to the injured region, fuel transcription of pro-inflammatory mediators [24] and act as a bridge for the initiation of the adaptive immune response [25]. However, pro-inflammatory mediators do not tell the whole story about macrophages.

In this review, we give an overview about general aspects of macrophages including their origin, classification, polarization markers and plasticity. We then focus on the evolving role of macrophages in acute allograft rejection, which ranges from pro-inflammatory to tolerogenic, and on the impact of clinically used immunosuppressive drugs on macrophage function. Finally, we review emerging results on tetrahydrobiopterin (BH4), a vitamin-like compound readily synthesized in the human body and cofactor to eight vital reactions, which has been shown to impact ischemia reperfusion injury (IRI), acute rejection as well as chronic rejection following solid organ rejection, rendering it a potentially novel immunosuppressive agent addressing the innate immune system.

## 2. Types of Macrophages, Polarization and Markers

### 2.1. Tissue-Resident Macrophages (TRM) and Monocyte-Derived Macrophages

Macrophages (large eaters, from Greek: μακρός and φαγεῖν) are white blood cells that are part of the innate immune system and their specialty is to detect and destroy pathogens [26,27]. In the body, two distinct pools of these cells exist, i.e., monocyte-derived macrophages that originate from bone marrow and tissue-resident macrophages (TRM) that arise during fetal development. The latter derive from the yolk sac and the fetal liver [28] and can generate specific phenotypes depending on the organ where they are located [29]. In the liver, they can be found as Kupffer cells, whereas bone-located macrophages are called osteoclasts and microglia are resident macrophages in the brain [22]. Additionally, the gut presents with resident macrophage populations that have distinct phenotypes and functions and are crucial for maintaining tolerance to the gut flora and orally administered antigens [30,31]. In the spleen, a special subtype of resident macrophages suppresses immunogenic reactions to apoptotic cells by efficiently clearing their material [32] and macrophages lining the subcapsular sinus of lymph nodes are involved in capturing lymph-borne pathogens [33]. For a comprehensive review, describing these different types of TRM, including others such as Langerhans cells and alveolar macrophages, the reader is referred to [28]. In contrast to the TRM, monocyte-derived macrophages originating from the bone marrow are patrolling in the blood circulation as part of the peripheral blood mononuclear cells (PBMCs). They can adhere to the vascular endothelium and pass through the endothelial cell gap, migrate to the tissues and then develop locally into differently polarized macrophages [34].

### 2.2. Macrophage Phenotypes and Respective Markers

Back in the 1980s, interferon (IFN) γ produced by Th1 helper cells was found to activate macrophages [35], while the Th2 cytokine interleukin (IL)-4 was identified as a mediator of macrophage activation, which however led to a different phenotype as that observed with IFNγ, in the 1990s [36]. Finally, in 2000, nitric oxide (NO) and arginine production profiles were compared in two mouse strains, a prototypical Th1 (C57Bl/6) and Th2 strain (BALB/c), and the authors proposed the Th1-elicited macrophages to be called M1, while the Th2-elicited macrophages were since then terminated M2 [37]. Metabolic studies have recently shown that certain metabolic pathways are closely related to the phenotype and function of macrophages, as M1 macrophages display increased glycolysis, fatty acid synthesis (FAS) and pentose phosphate pathway (PPP) metabolism. In M2 macrophages, high mitochondrial oxidative phosphorylation (OXPHOS) can be found and enhanced fatty acid oxidation (FAO) and glutamine metabolism are linked to M2 activation (for detailed information the reader is referred to [38]).

Phenotypically, macrophages are identified as CD45+ cells that express myeloid cell markers (general phenotypic marker for mice are, e.g., CD11b, F4/80, CD68, MERTK, and Ly6C; markers for humans are, e.g., CD11b, CD14, CD16, CD33, and CD68) but lack surface markers that are associated with adaptive immune cells (e.g., CD3, CD19, CD56, NKp46, TCR, and BCR). Regulatory macrophages (Mregs) in mice additionally express CD11c, CD169 and Dectin-1 [39], while CD11c [40] and DHRS9 [41,42] are found in humans. It should be emphasized that macrophages also express receptors for chemokines, cytokines, DAMPs, Fc fragments and complement products. These receptors empower macrophages to respond to a broad bundle of immune and inflammatory mediators as a result of complement activation, tissue inflammation, T-cell activation, antibody production, as well as dysregulated cell death [41]. Hence, the expression of such additional receptors further divides macrophages into complex subsets, especially in relation to their activation status, anatomical location or division of functional activities. Table 1 gives an overview of the so far described main monocyte-derived macrophages and TRM concerning their corresponding functional characteristics and markers.

M1 macrophages (also named classically activated or pro-inflammatory macrophages) can be induced by Th1 cytokines such as IFNγ and tumor necrosis factor (TNF) α as well as microbial products or DAMPs. From a molecular point of view, the induction by these factors leads to the activation of signal transducer and activator of transcription (STAT) 1, interferon-regulatory factor (IRF) 5 and, nuclear factor kappa B (NF-κB), which drive the expression of M1 markers such as inducible nitric oxide synthase (iNOS), MHC class II and inflammatory mediators such as IL-1β, IL-6, IL-12, IL-23, TNFα and cyclooxy-genase (COX) 2 [57]. Functionally, M1 macrophages play a role in the removal of pathogens during infection, harbor anti-microbial activity, mediate reactive oxygen species (ROS)-induced tissue damage and impair tissue regeneration and wound healing [27].

M2 macrophages (also named alternatively activated, anti-inflammatory macrophages) are activated by IL-4 or IL-13 which by binding to the IL-4 receptor activate STAT6 and IRF4 that drive transcription of M2 markers such as arginase (ARG) 1, peroxisome proliferator-activated receptor (PPAR) γ, suppressor of cytokine signaling (SOCS) 1, CD163, CD206, anti-inflammatory mediators such as IL-10 and transforming growth factor (TGF) β as well as other growth factors such as vascular endothelial growth factor (VEGF), platelet-derived growth factor (PDGF) and insulin-like growth factor (IGF). M2 macrophages are involved in, e.g., fibrosis, angiogenesis, wound healing, and phagocytosis of dying cells, and usually, the phenotype of TRM reflects such an anti-inflammatory M2 state [57]. The M2 phenotype can be further subdivided into type 2a (induced by IL-4 and IL-13, promoting fibrosis), type 2b (induced by immune complexes (ICs), toll-like receptor (TLR) agonists or IL-1 receptor ligands, producing pro- and anti-inflammatory cytokines), type 2c (induced by IL-10 and glucocorticoids (GC), inhibiting inflammation and eliminating apoptotic cells via phagocytosis), and type 2d (activated by TLR ligands through A2 adeno-sine receptor (A2R) agonists, inducing anti-inflammatory cytokines, favoring angiogenesis, features similar to tumor-associated macrophages (TAMs) (for a review, see [27,58]).

### 2.3. Macrophage Plasticity

In recent decades, the original classification of merely M1, M2 and M2 subtypes, which arose mostly from in vitro experiments and does not represent the actual in vivo situation, was challenged by the emergence of more and more phenotypes lying in between the two polarization extremes, M1 and M2 (see Table 1). As a clear and comprehensive nomenclature for the variety of known macrophage states was missing, a fact that greatly impacts on the possibility to compare the data of different published studies, there have been efforts to implement a more universal nomenclature. A suggestion to better describe the remarkable and interchangeable heterogeneity of polarization phenotypes was published in 2014 which postulated to include source (e.g., macrophages generated in vitro from human peripheral blood or murine bone marrow and macrophages isolated from human or murine tissues) and conditions used for macrophage polarization/activation as well as the activation agent (e.g., M(IL-4), M(IL-10), and M(IFNγ)) [53].

Even once polarized to a certain subtype, macrophages remain highly plastic in vivo and can quickly readapt their phenotype depending on the surroundings and the demand of the tissue they are located in [59]. Due to their highly plastic properties, macrophages can assist in all steps of inflammation starting from the initial inflammatory mounting phase and ranging to the resolution phase where tissue regeneration and repair is finalized [60]. In a zebrafish fin amputation model, it could be shown that macrophages were attracted to the wound in a single wave and changed their shape and behavior depending on the requirements directly at the wounded site [61]. The same study also provided a link to lipid metabolism, by showing the implication of polyunsaturated fatty acid (PUFA) metabolism and 15-lipoxygenase on the morphology and polarization of macrophages. In macrophages harvested from either aged or tumor-bearing mice, it was shown that they could be reprogrammed by exposing them to a different cytokine environment [62]. Still, the exact mechanisms leading to reprogramming and adaptation are not yet fully understood. As macrophage plasticity is important also for other immunological challenges such as organ transplantation [58] and disturbances in macrophage plasticity can lead to pathogenic conditions such as cancer, asthma and atherosclerosis, there is an urgent need to better understand the underlying processes at a molecular level.

## 3. Macrophages in Acute Rejection and Organ Tolerance

In contrast to the adaptive immune system entailing T-cell- and B-cell-mediated immunity, the role of the innate immune system is still less appreciated in allograft recognition. This, despite the already mentioned observation, that macrophages represent up to 60% of cellular infiltrates of the allograft during the rejection process [20] and that the amount of graft infiltrating macrophages has been correlated with worse outcomes [63].

The peak time of infiltrating monocyte-derived cells occurs within the first 48 h after reperfusion of the transplanted organ, with M1 macrophages initially dominating the scene. Three to five days later another phenotype predominates the graft, the M2 macrophages [64]. M1 macrophages are involved in tissue injury as a response to damage and pro-inflammatory signals while M2 macrophages mediate tissue remodeling and injury resolution, suggesting them as promoters of graft damage repair [21]. M2 macrophages might however also prevent and not only resolve the damage. In murine corneal allografts, an increase in M1 macrophages was observed during acute rejection while M2 macrophages were present in those mice that did not reject the transplant [65]. However, the simplified discrimination into classically activated M1 macrophages induced by Th1 cytokines presenting pro-inflammatory properties and alternatively activated M2 macrophages polarized by Th2 cytokines harboring anti-inflammatory and immunoregulatory properties is often challenging as switches between both macrophage phenotypes occur [27]. This complexity based on interactions of macrophages with their environment is also reflected in the different roles they assume during the entire process of allograft engraftment. This starts with IRI of the transplanted organ, passes over to acute allograft rejection and eventually ends in chronic allograft rejection characterized by allograft vasculopathy and graft failure (for a comprehensive review, we refer readers to [58]).

### 3.1. M1 Macrophages

It is nowadays accepted that the innate and the adaptive immune system interact with each other, with the former acting as a bridge to the activation and initiation of the latter resulting finally in allograft rejection. The basis for the injury promoting deleterious processes in the transplanted grafts starts already before the implantation of the graft. The brain death of the donor, the retrieval of the organ with its associated ischemia (which is even worse in case of donations after cardiac death) and the subsequent reperfusion of the organ result in the so-called IRI [66].

This initial injury triggers several processes promoting infiltration of mononuclear phagocytic cells with a predominant pro-inflammatory M1 phenotype. Crucial is the occurrence of tissue injury-associated DAMPs, which are recognized and processed by these innate immune cells thanks to PRRs such as TLR, NOD-like receptors or C-type lectin receptors, resulting in increased transcription of pro-inflammatory mediators. In addition, the increased expression of the transcription factor hypoxia-inducible factor 1α (HIF1α) induced by tissue hypoxia also triggers increased expression and production of pro-inflammatory cytokines. As a result, the classically activated macrophages that predominate in the initial phase generate high amounts of ROS and reactive nitrogen species (RNS) [67] aggravating allograft injury and secrete pro-inflammatory cytokines such as IL-1β, IL-12, IL-18, TNFα and IFNγ [68,69,70,71,72]. This results in activation of endothelial cells and promotion of cytotoxic T-cell generation [73,74]. The pivotal role of macrophages during acute allograft rejection is not only suggested by the presence of macrophages in specimens showing acute cellular rejection [75] but also by transcriptome analyses of such biopsies. In the latter, prominent transcript levels of the macrophage colony-stimulating factor (M-CSF) are noteworthy since this chemokine produced by the injured renal tubular epithelial cells (TEC) induces increased surface expression of MHC class II (in humans HLA-DR) and co-stimulatory molecules CD40 and CD80 on monocytes transforming them into antigen presenting cells (APC) [76]. Still, the initiation seems to be based on promoting cell differentiation of activated T-cells rather than antigen presentation since macrophages are weak antigen presenters compared to dendritic cells [77]. In fact, alteration of surface molecules such as phosphatidylserine receptor TIM4 or DC-SIGN (also known as CD209) can switch their ability from promoting differentiation of effector or helper T-cell to enhance the expansion of CD4+CD25+FoxP3+ regulatory T-cells (Tregs) [78,79]. Similarly, depending on the secreted cytokines macrophages might promote an allograft rejecting Th1/Th17 [80] or an allograft accepting CD4+CD25+FoxP3+ environment [40].

Recent findings challenge the traditional view of macrophages as innate immune cells mounting a non-allogeneic-specific inflammatory reaction. In mice lacking adaptive immune cells, rejection of the allograft resulted in an innate immune response based on the activation of the CD47/SIRPα pathway. SIRPα is a receptor of the immunoglobulin superfamily that mediates a “do not eat me” signal to docking macrophages. In this non-MHC-dependent allorecognition, SIRPα polymorphism of the donor was recognized by infiltrating macrophages allowing them to discriminate between self and non-self and to further start the rejection response [40,81].

Another finding challenging the traditional view of innate immune cells is the “trained immunity”. Macrophages can not only sense non-self antigens, but they might also develop immune memory. In a mouse model, macrophages exposed to allogeneic stimuli on day zero were able to develop an elevated immune response up to four weeks after the first exposure. The allogeneic memory was mediated by the paired immunoglobulin-like receptors-A (PIR-A) receptor binding the non-self MHC class I antigen and blocking this receptor resulted in prolonged allograft survival [82,83].

In line with these findings, in a rat kidney transplantation model of T-cell-mediated acute rejection, depletion of macrophages with liposomal-clodronate or c-fms kinase inhibitor improved allograft function attenuating its injury [84,85].

However, rather than just unspecifically deleting the presence of macrophages, shaping polarization of macrophages emerges as another strategy to prevent organ rejection.

### 3.2. M2 Macrophages

The role of M2 macrophages in acute rejection is heavily discussed as well as whether targeting macrophages in transplant immunology would present a promising treatment approach [78,86,87]. As they are able to amplify (anti-)inflammatory processes by cross-talking with the adaptive immune system, some therapeutic approaches of macrophage manipulation have recently moved into the focus of transplant immunology research [88,89]. As already mentioned above, M2 macrophages play a janus-faced role in solid organ transplantation. While playing an anti-inflammatory role, limiting excessive tissue injury caused by the initial infiltration of the predominantly M1 phenotype, M2 macrophages are crucial in the development of chronic allograft rejection promoting allograft fibrosis and chronic allograft vasculopathy.

Regarding acute rejection, there are different modulatory effects attributed to alternatively activated M2 macrophages. In mice, one of these effects could be related to the high expression of the M2-associated enzyme ARG1. Consumption of L-arginine leads to reduced T-cell proliferation and effector response [90]. Next, production of anti-inflammatory cytokines such as IL-10 promotes induction and proliferation of FoxP3+ Tregs [78]. Additionally, also increased phagocytosis has been recently observed to modulate acute rejection. Treatment with mesenchymal stem cells (MSC) overexpressing soluble fibrinogen-like protein 2 (sFgl2) which is mainly produced by Treg cells significantly delayed acute rejection and promoted the polarization of M2 macrophages in the graft. These M2 macrophages showed a higher migration and phagocytosis capacity in vitro when treated with IFNγ and lipopolysaccharides (LPS), suggesting a high activity in resolving the existing damage [87]. With regard to MSC, in a mouse transplantation model, local co-application of MSC and CsA resulted in a phenotype switch of infiltrating macrophages from M1 to M2. This was characterized by a decrease in intragraft NO and IFNγ production, while alternatively activated F4/80+ CD206+ M2 macrophages and IL-10 became predominant [91].

In a mouse cardiac transplantation model, tolerance acquired by co-stimulatory blockade with anti-CD40L mAb resulted in a conversion of pro-inflammatory macrophage precursors to M2-like regulatory macrophages. Interestingly, depletion of CD11b expressing monocytes of the recipients prevented occurrence of tolerance [78,92]. Using the same tolerance model, the presence of M2 macrophages was associated with graft infiltrating neutrophils secreting higher levels of M-CSF, a growth factor known to regulate macrophage proliferation and differentiation [93].

### 3.3. Regulatory Macrophages (Mregs)

Allograft-specific tolerance of the recipient immune system is the holy grail of transplant medicine. Since lifelong maintenance of immunosuppression is associated with serious long-term complications, allograft-specific tolerance would allow to significantly reduce the need for pharmacological immunosuppression. Historically, these studies include depletion protocols aimed to generate hematopoietic chimerism and focused on the adaptive immune system [94].

However, recent data provide evidence that a subtype of macrophages, the so-called regulatory macrophages (Mregs), can also induce a tolerogenic environment. Mregs can be seen as a different state of polarization since they differ from both the M1 and the M2 subtypes (Table 1). They possess a strong antigen presenting capacity due to highly expressed CD80 and MHC class II receptors and harbor an anti-inflammatory property producing large amounts of IL-10 and TGF-β and low levels of IL-12. For an in-depth review, we would refer the readers Zhang and colleagues [95]. The immunosuppressive capacity of Mregs is described to rely on different mechanisms. One is the secretion of anti-inflammatory cytokines such as IL-4, IL-10 and TGFβ [96]. Using a tolerogenic mouse cardiac transplantation model based on anti-CD40L mAb administration, the presence of Mregs characterized by DC-SIGN expression was flanked by increased numbers of CD4+ CD25+ FoxP3+ Tregs, decreased CD8+ T-cell activity and prominent intragraft levels of IL-10 [78]. From a mechanistic point of view, direct delivery of inhibitors affecting the M1 polarization pathways mTOR and NF-κB via nanoparticles also resulted in shifting macrophages towards a suppressive phenotype reducing CD8+ T-cell-mediated alloreactivity and Treg expansion [97]. In vitro, Mregs have also been shown to phagocyte and inactivate allogeneic T-cells by direct contact in an iNOS-dependent fashion. A single administration of ex vivo-generated donor-derived CD11b+ Ly6C-/low Ly6G- CD169+ Mregs before transplantation (either eight or 35 days before transplantation) resulted in prolonged allograft survival of fully allogeneic heart transplantation in mice. Of note, third party or recipient-derived Mregs did not confer the observed protective effect [39]. In this regard, the missed benefit of postoperative administration of in vitro cultured CD14+, CD16+, and CD18+ Mregs in a porcine lung transplantation model points to the importance of a preoperative administration as an optimal time frame [98].

Mregs have already found their way into clinical trials. Early clinical trials using these cells showed technical feasibility and safety of this immunosuppressive strategy [99]; however, as for experimental studies, only the switch from a postoperative [100] to a preoperative application [101] of these cells led to successful minimization of the immunosuppression in some patients. The EU-funded consortium called The ONE study developed a range of cell-based medicinal products (CBMPs) thought to be administered to allograft recipients with the final goal to reduce standard-of-care maintenance immunosuppression. The six developed CBMPs included two polyclonal Tregs (pTreg-1 or pTreg-2), two donor-antigen-specific Tregs, one tolerogenic dendritic cell and one Mreg cell product. Of note, all regulatory cell products except Mregs were derived from the recipient. The Mregs were the only donor-derived cell product. Phase 1/2A trials in living-donor kidney recipients using these CBMPs showed similar incidences of biopsy-proven acute rejections compared to standard-of-care maintenance immunosuppression, successful reduction in maintenance immunosuppression in nearly half of the patients and fewer infectious complications [102]. From a mechanistic point of view, co-culture and humanized mouse experiments showed that donor-derived Mregs induced a high proportion of FoxP3- CD4+ T-cells to become IL-10 producing FoxP3+ Tregs. These macrophage-induced Tregs did not derive from natural (n)Treg developed in the thymus and expressed cell-surface T-cell immunoreceptor with Ig and ITIM domains (TIGIT). Of note, these TIGIT+ iTregs were found in a recipient of The ONE study receiving Mregs (ONEmreg12 trial) at the seven year follow-up post transplantation and might be crucial in contributing to long-term acceptance of the graft. Among the multiple non-redundant molecules involved in TIGIT+ iTregs development also indoleamine-2,3-dioxygenase (IDO) and progestagen-associated endometrial protein (PEAP) were identified, two crucial players in fetal tolerance [40,103,104]. Even though the outcomes following Mregs infusion in human transplant recipients are still unclear, they clearly point at the importance of donor-derived macrophages in orchestrating allograft rejection and at the necessity to intervene in this deleterious process before antigen presentation in the recipient occurs.

Another promising approach is the use of mediators secreted by macrophages during efferocytosis. In a model of collagen-induced arthritis, injection of the supernatant from macrophages that are eliminating apoptotic cells (SuperMApo) resulted in resolution of ongoing inflammation. This was supported by in vitro data showing that co-culturing APC with SuperMApo triggered their return to homeostasis downregulating the molecular expression of co-stimulatory molecules. Of note, this resolution of inflammation was antigen-specific, and animals treated with SuperMApo were still competent to reject skin allografts [105]. Pro-resolving mediators in chronic diseases are already in clinical trials to test their safety and efficacy for the signs and symptoms of dry eyes as well as treatment of gingivitis (NCT00799552 and NCT02342691, available on the clinicaltrial.gov website).

### 3.4. Tissue-Resident Macrophages (TRM)

Residing in different organs, TRM further highlight the complex heterogeneity of macrophages. Osteoclasts in the bone, Kupffer cells in the liver or microglia in the brain are tissue-specific macrophages with the specific function to maintain tissue homeostasis and normal organ function. In mice, TRM are generally considered to have immunosuppressive function for maintaining tissue homeostasis, inhibiting T-cell activation and resolving ongoing inflammatory processes [106,107]. As a result, macrophages identified in injured organs derive from two different sources, TRM and circulating monocytes [108]. Unique to allograft transplantation is the fact that donor TRM residing in the transplanted graft are transferred to the recipient.

In this regard, the role of TRM is not clearly defined. Still, there is important data pointing at a steering role of TRM. In a mouse cardiac transplantation model, a subset of TRM expressing high levels of CD169 and TIM4 was shown to migrate to the draining lymph nodes inducing antigen-stimulated Tregs and finally promoting cardiac allograft engraftment [109]. On the contrary, in the lung, severe IRI has been observed to trigger increased TLR4 expression on alveolar macrophages, resulting in increased endotoxin levels and neutrophil recruitment in the donor organ aggravating allograft injury [110,111].

These different outcomes could be related to different TRM populations present in distinct organs. In the human heart, TRM can be distinguished by the expression of CCR2. While CCR2+ macrophages derive from adult hematopoietic progenitor cells, depend on circulating monocytes for maintenance and are involved in pro-inflammatory processes recruiting regularly monocytes and neutrophils, CCR2- macrophages derive from embryogenic progenitors, are independent of circulating monocyte replenishment and promote tissue repair [112]. In a recently published study using a murine heart transplantation model, donor CCR2+ macrophages and MYD88 signaling were identified as crucial drivers of allograft rejection. Depletion of either donor CCR2+ macrophages or MYD88 signaling resulted in prolonged allograft survival, in contrast to the deletion of donor CCR2- macrophages which resulted in a more rapid allograft rejection and cellular infiltration. Of note, targeting recipient CCR2+ macrophages did not show any survival benefit and the application of CTL4-Ig immunosuppression preserved donor CCR2- but not CCR2+ macrophages [113]. Hence, similarly to the polarization of infiltrating macrophages, there also seems to exist a fragile equilibrium between TRM. The exponential development and use of ex vivo normothermic perfusion techniques could represent the ideal platform to further pursue TRM as treatment target [114,115].

Another aspect underlining the complexity of TRM is the role of phagocytosis-mediated clearance of either injured graft cells or infiltrating lymphocytes. During renal injury, renal TEC transform themselves into semi-professional phagocytes expressing kidney injury molecule 1 (KIM-1) and are capable of clearing apoptotic cells [116]. In a murine model of kidney transplantation, the improvement of the phagocytic activity from TEC by the administration of recombinant apoptosis inhibitor of macrophages (rAIM) marked-ly reduced the delay of graft function. A single dose of rAIM resulted in almost normal kidney function within 48 h post-transplantation with reduced tissue inflammation and necrotic debris [117]. In the same line, in a mouse liver transplantation model, the promotion of Kupffer cell clearance capacity from apoptotic T-cells resulted in a reduced rejection activity index. The improved liver graft function was associated with an M2 polarization of Kupffer cells triggered by PUFAs from degraded T-cells which in turn activates PPARγ [118].

## 4. Effect of Maintenance Immunosuppressive Agents on Macrophages

Lifelong immunosuppression constitutes the pillar of long-term success in solid organ transplantation; however, the agents used for maintenance immunosuppression are hampered by drug toxicity and adverse effects are harming patient as well as graft survival [7]. The effect of immunosuppressants on T-cells has been extensively studied in recent years as T-cells were assumed to be the main immunological contributors of transplant rejection. However, their effect on innate immune cells, especially macrophages, which increasingly move into the focus of transplant rejection, remains limited and poorly investigated. In this chapter, we summarize recent findings to gain a better insight into the effects of maintenance immunosuppressants that are currently used in clinics including the mammalian target of rapamycin (mTOR) inhibitor rapamycin, the two calcineurin inhibitors CsA and tacrolimus, mycophenolate mofetil (MMF), GC as well as belatacept on macrophages [119]. An overview about the main effects of these immunosuppressives can be found in Table 2.

### 4.1. mTOR Inhibitor (Rapamycin/Sirolimus)

Rapamycin, also known as sirolimus, is an immunosuppressive drug that inhibits mTOR, a serine-threonine kinase involved in cell growth, protein synthesis, proliferation and apoptosis [149]. mTOR inhibitors are known to suppress T-cell proliferation thereby influencing graft rejection [150].

Studies investigating the role of mTOR inhibition on macrophages in a severe combined immunodeficiency (SCID) model showed that treatment of T-/B-cell-deficient mice and immunocompetent C57Bl/6 mice with rapamycin as well as mTOR knockout decreased the levels of CD11b+ F4/80+ macrophages. This impaired macrophage/monocyte development during mTOR deficiency goes in hand with an overexpression of STAT5 and further downregulation of IRF8 that downregulates M-CSF/CD115 signaling which is important for the differentiation and proliferation of monocytes/macrophages [120].

Additionally, in a murine heart transplantation model with antibody-mediated rejection, mTOR inhibitors were found to attenuate macrophage infiltration as well as transplant allograft vasculopathy [121]. Furthermore, rapamycin treatment in human macrophages polarized to M1- and M2-like phenotypes was found to induce apoptosis exclusively of M2 macrophages and the cytokine profile of TLR4-stimulated PBMCs from rapamycin-treated patients prior islet transplantation showed a shift towards a M1-like macrophage phenotype [122]. Such influence on M2-like macrophages was also found in bone marrow-derived macrophages with mTOR deletion in which M2 polarization was inhibited. Macrophage-specific knockout of mTOR in a murine heart transplantation model had no influence on acute allograft rejection while additional treatment of mTOR-deficient mice with the immunosuppressive agent CTLA4-Ig inhibited chronic rejection and expanded Foxp3+ Tregs in allografts [123]. In another murine heart transplantation model, the treatment of an mTOR-specific high-density lipoprotein (HDL) was shown to prevent aerobic glycolysis in macrophages and epigenetic modifications for the production of inflammatory cytokines. Simultaneously, the development of Mregs was induced which inhibited alloreactive CD8+ T-cell proliferation and expanded immunosuppressive Foxp3-expressing Tregs [97]. A reduction in inflammatory cytokines by rapamycin was also found in vitro in a human monocyte cell line as well as in primary monocytes from humans, in which LPS-induced chemokine expression of monocyte chemoattractant protein 1 (MCP-1, CCL2), IL-8, RANTES (CCL5), macrophage inflammatory protein (MIP) 1α and MIP-1β was decreased after rapamycin treatment [124].

All these findings point to a strong effect of rapamycin on macrophages by impairing their development, inhibiting the polarization to a M2-like phenotype in the setting of chronic rejection, decreasing their release of inflammatory cytokines and boosting the formation of Mregs.

### 4.2. Calcineurin Inhibitors (Cyclosporine A and Tacrolimus)

Calcineurin inhibitors, such as CsA and tacrolimus, are immunosuppressive drugs that act through inhibition of the calcineurin pathway, which decreases expression of IL-2 and other cytokines in T-cells, inhibits NF-κB activation and impairs T-cell activation, proliferation and differentiation [151,152].

A consequence of tacrolimus treatment on macrophages was identified in a rat model of spinal cord injury where the treatment led to lower TNFα pro-inflammatory cytokine production and M1 marker expression than in controls [125]. Additionally, polarization of PBMCs indicated a shift to M2-like surface marker after tacrolimus treatment of blood samples while IL-1β production and phagocytosis function of macrophages were not altered [126]. Tacrolimus-treated IL-10-deficient mice presented with lower pro-inflammatory cytokine production, suppressed LPS-induced activation of both NF-κB and MAPK in peritoneal macrophages and induced apoptosis of macrophages via the activation of caspases 3 and 9 [127]. Furthermore, in a urinary tract infection model, tacrolimus pretreated mice displayed higher bacterial loads than control mice. This impaired host innate immune response after tacrolimus treatment was characterized with reduced TLR5 expression in bladder macrophages [153]. In accordance with these findings, macrophages treated with tacrolimus in vitro showed a decreased responsiveness to LPS induction and macrophages from tacrolimus-treated mice or mice with myeloid-specific calcineurin B1 (CnB1) knockout had diminished LPS-induced inflammatory responses [128]. Additionally, in human monocytes/macrophages, CsA reduced NF-κB activation and dose-dependently inhibited the LPS-induced procoagulant activity, which was identified as tissue factor activity associated with coronary lesions and fibrin deposition [129]. In a rat kidney transplantation model, CsA treatment led to increased macrophage infiltration and lower graft survival when compared to a CCR5/CXCR3 antagonist treatment [154]. Moreover, such an accumulated macrophage influx together with increased interstitial fibrosis was seen in kidneys of rats suffering from CsA-caused nephrotoxicity [155].

Comparison of CsA and tacrolimus in rat renal allografts indicated that tacrolimus treatment improved changes in acute and chronic rejection and additionally inhibited the fibrogenic PDGF, while moderate to intense histological changes as well as an induction of PDGF were identified upon CsA treatment [130]. Consistent with this finding, renal biopsies from CsA-treated patients during chronic rejection showed increased tubulointerstitial CD68-positive macrophages together with a lower graft survival rate compared to tacrolimus-treated biopsies [131]. A clinical trial also showed that CsA treatment led to increased interstitial fibrosis as well as total cholesterol and low-density lipoprotein (LDL) levels while tacrolimus treatment showed a trend for insulin resistance after renal transplantation. However, no difference in the incidence of acute rejection in both treatments were identified [132].

In general, both calcineurin inhibitors affect macrophages by decreasing their NF-κB pathway activation and response to LPS with tacrolimus leading to a decrease in M1 surface markers with a shift to a M2-like phenotype and reduced production of pro-inflammatory cytokines. Of note, in the setting of chronic rejection, CsA treatment is characterized by increased macrophage infiltration and fibrosis compared to tacrolimus.

### 4.3. Mycophenolate Mofetil (MMF)

The immunosuppressive compound mycophenolate mofetil (MMF) is the ester prodrug of mycophenolic acid (MPA), an inhibitor of the enzyme inosine-5′-monophosphate dehydrogenase which is involved in nucleotide synthesis. MPA was shown to deplete guanosin nucleotides in T- and B-cells thereby inhibiting their proliferation and suppressing cell-mediated immune responses [156].

A possible effect of MPA on macrophages has already been assumed in inhibited synthesis of BH4, which is built from guanosine triphosphate (GTP). BH4 is the cofactor of several enzymes including iNOS, an enzyme involved in the inflammatory response of macrophages [157].

In MRL/lpr mice, a genetic model of generalized autoimmune disease, the treatment of dextran mycophenolate-based nanoparticles lowered the levels of pro-inflammatory mediators and reduced kidney injury. Additionally, these MPA nanoparticles were found to be phagocytosed in the spleen and kidney by macrophages which were characterized as CD206+ M2-like phenotypes with downregulated CD80 and CD40 surface markers and reduced TNFα production [133]. Likewise, treatment of PBMCs with MPA was also shown to have no influence on the phagocytosis function of macrophages but IL-1β production was reduced to 50% and an increase in M2 surface markers such as CD163 and CD200R on M1-polarized macrophages could be identified [126]. In addition, LPS-stimulated monocytes derived from renal transplant recipients that were treated with MMF showed decreased secretion of IL-1β together with lower IL-10 and TNFα secretion, reduced TNF-R1 expression and suppressed antigen presenting capacity [134]. Moreover, MMF-treated human monocytes presented with reduced binding to endothelial cells, inhibited upregulation of intracellular adhesion molecule 1 (ICAM-1) and decreased MHC-II expression on monocytes that were stimulated with either LPS or IFNγ [135].

Overall, MPA influences macrophages by promoting an M2-like macrophage phenotype, decreasing pro-inflammatory mediators and lowering the interactions of monocytes/macrophages and endothelial cells.

### 4.4. Glucocorticoids (GC)

GC are steroid hormones that are widely used due to their anti-inflammatory and immunomodulatory properties. They are involved, e.g., in the stabilization of lysosomal membranes, the suppression of prostaglandin synthesis and reduction in histamine release [150,158]. GC bind to their glucocorticoid receptor (GR), resulting in inhibition of transcription and production of several pro-inflammatory cytokines such as IL-1, IL-2, IL-6, IFNγ and TNFα by several complex steps [150].

A possible influence of GC on macrophages was speculated decades ago due to a side effect of GC treatment leading to reduced bone density where GC were found to directly act on bone marrow-resident macrophages, namely osteoclasts [60,159]. In a mouse model of contact allergy, the ablation of the GR in macrophages abolished downregulation of the inflammatory cytokines and chemokines such as IL-1b, MCP1, MIP2 and IFNγ-inducible protein 10 (IP10) by GC while its inactivation in keratinocytes or T-cells did not attenuate the effects of GC [136]. In the same line, macrophage-specific GR-deletion in a mouse model of induced colitis presented with increased macrophage infiltration at inflammatory sites in the colon as well as pro-inflammatory cytokine expression while expression of scavenger receptors and IL-10 were diminished [160]. Treatment of the macrophage cell line J774 with dexamethasone or hydrocortisone was found to cause a concentration-dependent inhibition of NO formation, suggesting an inhibition of nitric oxide synthases [137]. In contrast, the administration of corticoids during transient cerebral ischemia were found to increase the enzymatic activity of endothelial nitric oxide synthase [161]. However, in macrophages a dose-dependent effect of GC on NO production characterized by enhanced NO production after administration of low corticosterone levels concomitant with increased expression of pro-inflammatory cytokines and enzymes for mediator synthesis were identified while high doses of corticosterone strongly repressed macrophage functions [138]. In human kidney allografts with antibody-mediated or T-cell-mediated rejection, steroid treatment increased macrophage infiltration into the allograft, which was additionally accompanied with increased cell proliferation and antigen presentation [139]. Furthermore, prednisolone treatment of mice with mesangial proliferative glomerulonephritis was found to induce a M2-like macrophage phenotype and intensified glomerulosclerosis [140]. Similarly, GC treatment in human monocyte-derived macrophages induced differentiation of a specifically activated, anti-inflammatory subtype which enhances phagocytosis and motility as well as repression of adhesion, apoptosis, and oxidative burst [141]. In addition to the effect of GC to promote phagocytosis, they were also identified to enhance bacterial killing in vitro [142] and enhance CD163 expression and production of TGFβ1, fibroblast growth factor 2 (FGF-2), connective tissue growth factor (CTGF) indicating that steroids mimic a pro-fibrotic phenotype [143].

In summary, GC act on macrophages by enhancing pro-fibrotic, M2-like macrophage phenotypes and promoting phagocytosis while downregulating pro-inflammatory cytokines.

### 4.5. Belatacept

Belatacept is a second-generation fusion protein combining the extracellular domain of the human cytotoxic T-lymphocyte-associated protein 4 (CTLA-4) with the Fc domain of human immunoglobulin G1 (CTLA4-Ig). It binds to the co-stimulatory molecules CD80 and CD86 on APCs thereby preventing T-cell binding to these molecules and inhibiting T-cell activation [162]. There is an obvious link between belatacept and macrophages as this compound blocks CD80/86 co-stimulatory molecules on APCs, which also include macrophages [163]. A study of renal transplant biopsies from patients treated with either calcineurin inhibitor or belatacept indicated that co-stimulatory blockade with belatacept was positively associated with genes related to innate immune responses including receptors of phagocytes (e.g., CD16, CD14, CD68, CD209, and TLR4) [144]. However, the effect of belatacept on macrophages is not well investigated. Nevertheless, there is evidence that CTLA4-Ig are affecting macrophages, which was already shown for abatacept, a first-generation CTLA4-Ig that is frequently used in the treatment of patients with rheumatoid arthritis. Bonelli and colleagues could show that CD14+ monocytes isolated from human PBMCs showed reduced migration capacity after treatment with abatacept [145]. Additionally, abatacept was found to reduce the production of pro-inflammatory cytokines such as TNFα, IL-12 and IFNγ in human macrophages generated from PBMCs [146]. Similarly, Cutolo and colleagues detected decreased IL-6, TNFα, IL-1β and TGFβ cytokine production in synovial macrophages from patients with rheumatoid arthritis compared to controls [147]. A recent study by Cutolo and colleagues could also show in monocyte-derived macrophages from peripheral blood that abatacept treatment in vitro induced the shift from M1 macrophages to M2 macrophages in LPS-induced macrophages from healthy subjects as well as macrophages obtained from patients with rheumatoid arthritis [148]. In general, an effect of belatacept on macrophages can be assumed as belatacept differs from its first-generation variant abatacept in two amino acid substitutions thereby leading to a higher affinity in binding to the co-stimulatory molecules [164].

## 5. BH4 as Immunosuppressive Player in Transplantation

In the last decade, we and others described BH4 in several animal studies to modulate the immune response in the setting of allograft transplantation. BH4 is a vitamin-like compound produced by probably almost every cell in higher organisms. It is an essential cofactor for a group of eight enzymes including (i) the three nitric oxide synthases (NOS), i.e., neuronal NOS (nNOS), inducible NOS (iNOS) and endothelial NOS (eNOS); (ii) four aromatic amino acid hydroxylases, i.e., tryptophan hydroxylases (TPH) 1 and 2, phenylalanine hydroxylase (PAH) and tyrosine hydroxylase (TH); and (iii) alkylglycerol monooxygenase (AGMO). These enzymes play a key role in several processes linked to cardiovascular function, neurotransmitter function and immune responses. Biosynthesis of BH4 occurs either via a de novo pathway requiring three enzymes, i.e., GTP-cyclohydrolase 1 (GCH1), 6-pyruvoyl tetrahydropterin synthase (PTPS) and sepiapterin reductase (SR), with GCH1 representing the rate-limiting enzyme or via a salvage pathway. In the latter, SR converts sepiapterin to 7,8-dihydrobiopterin which is finally transformed to BH4 by dihydrofolate reductase (DHFR) [165]. Several studies on IRI in transplantation and non-transplantation models observed a critical role of BH4 in preventing severe injury and loss of organ function. In a Langendorff perfused heart model, the endothelial-specific knockout of GCH1 led to a loss of BH4 in endothelial cells, resulting in endothelial dysfunction and increased cardiac infarct size following ischemic injury [166]. In the same line, supplementation with BH4 or its precursor sepiapterin reduced ischemic injury in rat models of cardiac infarction [167,168]. Implementation to a transplantation model revealed a similar protective effect in a murine pancreas transplantation model. Comparison with other pteridin-analoga showed that the protective effect was not due to the antioxidative capacity of BH4 but rather BH4-specific [169] and using knockout mice of the three different NOS isoforms revealed nNOS as BH4 target [170]. Similarly, treating the donor animal with BH4 resulted in a significant reduction in neointima formation in an aortic transplantation model. In this setting, BH4 supplementation was suggested to target eNOS preventing its uncoupling and the subsequent production of superoxide [171]. The importance of having saturated BH4 already before the major injury occurs was also observed in two ischemia preconditioning models where the increased expression of BH4 was clearly related to the protective effects of ischemic preconditioning [172,173].

With different groups describing a crucial role of iNOS in allograft transplantation, we tested in a murine allograft transplantation model the selective iNOS inhibitor 4-amino-BH4 (4-ABH4), the NOS-cofactor BH4 as well as the non-pterin inhibitor of iNOS N-(iminoethyl)-L-lysine (L-NIL). Only the two pteridines 4-ABH4 and BH4 resulted in prevention of allograft rejection while L-NIL did not affect the immune response, suggesting a pteridin-specific, iNOS-independent immunosuppressive effect of 4-ABH4 and BH4 [174]. Confirming this observation, overexpression of GCH1 was shown by another group to prevent allograft rejection pointing at an immunosuppressive role of pteridin compounds [175]. Of note, in this first publication demonstrating an immunosuppressive effect of BH4 in an acute rejection model, the microarray data showed that the repressed intragraft gene expression of CD163 during acute rejection was reversed by BH4 treatment [174]. This different expression in the M2 macrophage marker suggested that the effect might be related to the modulation of the innate immune system. In a follow-up study aimed at further dissecting the role of BH4 on the immune response during acute allograft rejection, we observed on day six following transplantation in BH4-treated animals increased levels of M2-related cytokines IL-10 and IL-4 in the graft as well as in the serum of the recipients. Of note, this was accompanied by a prominent mobilization of CD4+ CD25+ FoxP3+ Tregs in the spleen, a T-lymphocyte subpopulation known to be induced by alternatively activated macrophages (unpublished data). In the context of macrophages, BH4 has been mostly associated with iNOS since induction of this BH4-dependent enzyme is closely related to proinflammatory, classically activated macrophages [176]. However, a recent study using BH4-deficient Gch1fl/flTie2cre macrophages described BH4-dependent immune alterations that were not iNOS dependent. In particular, gene expression associated with the redox-sensitive transcription factor nuclear factor erythroid 2-related factor 2 (NRF2) were significantly reduced in BH4-deficient macrophages compared to wild-type ones [177]. Similarly, BH4-deficient Gch1fl/flTie2cre mice showed lower lung mycobacterial load following mycobacterial infection compared to wild-type animals, suggesting an anti-inflammatory role of BH4. Additionally, in this model, the effect could not be reproduced in mice lacking iNOS protein. In vitro quantification of mycobacterial growth confirmed the enhanced control of Gch1fl/flTie2cre macrophages compared to wild-type and iNOS-deficient cells [178].

**Figure 1:**
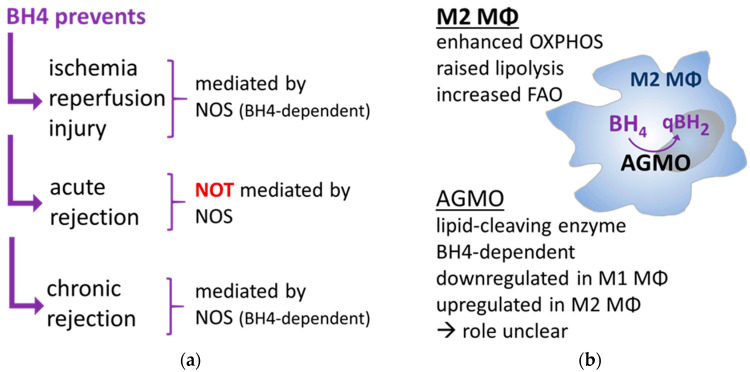
Immunosuppressive effect of BH4 in transplantation. (**a**) NOSs are identified as treatment targets for BH4-related immunosuppression in ischemia reperfusion injury and chronic rejection while NOS could be excluded as target for acute rejection. (**b**) Due to its upregulation in M2 macrophages that are linked with enhanced lipolysis, FAO and OXPHOS, AGMO might be the target for BH4-related immunosuppression in acute rejection.

Since iNOS, the hallmark molecule of M1 macrophages, has already been excluded as BH4 target in the context of acute rejection, the BH4-associated immunosuppressive effects might be related to another BH4-dependent enzyme, the recently described AGMO. AGMO is a lipid-cleaving enzyme that degrades ether lipids (especially alkylglycerols and lyso-alkylglycerophospholipids) into a free glycerol and a toxic fatty aldehyde, which is immediately converted to the corresponding fatty acid [179]. Ether lipids are a class of less well-studied lipids that harbor an ether bond at the *sn-1* position of the glycerol backbone compared to the commonly known ester lipids. Similar to their ester counterparts, ether lipids can also be found in biological membranes and can act as immune-related signaling molecules [180,181]. For AGMO, the (patho)physiological role has not yet been fully elucidated; however, there is increasing evidence that AGMO is involved in immunological processes [182]. In general, AGMO gene expression was found to be upregulated in M2 macrophages while it is strongly downregulated in M1 macrophages [183]. Of note, the rate-limiting enzyme in BH4 biosynthesis, GCH1, is inversely regulated as it is upregulated in M1 (most probably to boost iNOS) but not in M2 macrophages [183] potentially causing suboptimal AGMO turnover rates. This assumption is also supported by observations in rat livers where iNOS had an about 200-fold higher affinity to BH4 (Km between 0.02 and 0.3 µM) compared to AGMO (Km = 2.6 µM), which had a similar affinity to BH4 such as PAH (Km = 2 µM) [184,185]. In this context, BH4 treatment in the acute rejection model might fuel AGMO in M2 macrophages leading to increased lipid degradation. Such an upregulation of a lipid-cleaving enzyme would also fit to the already described metabolism of M2 macrophages in which enhanced lipolysis leads to increased OXPHOS and FAO activating M2 macrophages [38]. An overview about the immunosuppressive effect of BH4 and its targets in transplantation can be seen in Figure 1.

Last but not least, treatment with the orally active synthetic BH4-analogon sapro-pterin dihydrochloride has been already approved by the FDA (US Food and Drug Administration) and the EMA (European Medicines Agency) for patients with BH4-deficient phenylketonuria [186]. Unraveling novel mechanisms of a compound which is already in clinical use makes BH4-related immunosuppression an appealing strategy. Repurposing of a clinically used drug would eliminate time-consuming pharmacological development and safety testing required for novel drugs.

## 6. Conclusions

In recent years, a substantial amount of knowledge has been gathered regarding the role of macrophages in organ transplantation. The observation that these cells occur in different phenotypes displaying either pro- or anti-inflammatory properties makes them an appealing target to prevent acute allograft rejection or even induce graft tolerance. So far, one might only speculate on the factors affecting polarization of infiltrating or resident macrophages during acute allograft rejection. Considering, however, the different injuries an organ encounters already before the implantation procedure and then during the allograft recognition processes the ultimate amount of damage itself as well as the time point of counteracting these damaging factors might be crucial for the balance between pro- and anti-inflammatory macrophages.

In this regard, a better understanding of the effects of currently used maintenance immunosuppressive agents on macrophage polarization might result in the development of newer treatment protocols. Dosages as well as different application time points could be based not only on their effect of infiltrating lymphocytes but also on their effect on plasticity of TRM and infiltrating macrophages.

In addition, with lifelong immunosuppression still jeopardizing long-term graft and recipient survival due to drug toxicities and adverse effects, the development of new immunosuppressive agents addressing innate immune cells are urgently needed. We propose herein BH4 as a promising candidate. Administration of this vitamin-like compound has already been shown to prevent acute allograft rejection, and there is increasing evidence that its immunomodulatory role is not associated with iNOS activity but presumably with another BH4-dependent enzyme expressed in macrophages, AGMO. Both these BH4-dependent enzymes are abundantly found in activated macrophages. However, they are differently upregulated, iNOS in classically and AGMO in alternatively activated macrophage phenotypes. Successfully addressing the balance between different phenotypes by BH4 supplementation might be the starting point for a new generation of immunomodulatory drugs.

## Figures and Tables

**Table 1:** Functional characteristics and markers of different macrophage types *.

MΦ Type	M1	Atypical	M2a	M2b	M2c	M2d	Mreg	TRM
**stimulation/activation**	IFNγ LPS GM-CSF DAMPs PAMPs	mycobacterial infection IL-33	IL-4 IL-13 fungal and helminth infection	ICs IL-1R LPS	IL-10 TGFβ GCs	IL-6 LIF adenosine	M-CSF IFNγ	tissue environment IL-4 IL-13 IL-10 C1q, C3b normal flora (?)
**functional characteristics**	CD16 CD32 CD64 CD68 CD80 CD86 CD115 CCR7 MHCII IL-1R TLR2 TLR4 COX2 SOCS3 MARCO iNOS IDO	CD163 CD206 MGL1 YM1 iNOS	CD163 CD206 CD209 CD200R IL-1R MHCII Dectin-1 P2x7R SR TGM2 DecoyR ARG1 YM1/2 FIZZ1 ALOX15 [43,44]	CD86 MHCII	CD163 TLR1 TLR8 IL-21R MERTK	VEGF	CD301 CD209 CD169 CD11a Podoplanin CD127 CD204 CD80 iNOS DHRS9 XCR1 CD123 CD163 CD169 CD172a CD204 CD206 CD304 MER IDO	Axl CD192 (CCR2) CD68 CD115 CD206 CD273 (PD-L2) CD369 (Dectin-1) MHCII CD163 MGL-1 CD209 ARG1 iNOS [45,46,47]
**cytokine secretion**	IFNγ TNFα IL-1β Il-6 IL-12 IL-23	TNFα IL-6 IL-10 IL-12	IL-10 TGFβ IL1-RA	IL-10 high IL-1 IL-6 TNFα	IL-10 TGFβ	IL-10 TGFβ VEGF IL-12 low TNFα low	IL-10 TGFβ	IL-10
**chemokine secretion**	CCL10 CCL11 CCL5 CCL8 CCL9 CCL2 CCL3 CCL4	CCL18	CCL17 CCL18 CCL22 CCL24	CCL1	CCR2 CXCL13 CCL16 CCL18 [48]	CCL5 CXCL10 CXCL16	CCL1 [49] CCR1	CCR2

* Red letters highlight markers for mice only, blue letters indicate specific human markers and black letters are used for markers present in both organisms. ALOX15, arachidonate 15-lipoxygenase; FIZZ1/RETNLA/RELMα, resistin-like molecule-alpha; IL-1RA, IL-1 receptor antagonist; LIF, leukocyte inhibitory factor; MARCO, macrophage receptor with collagenous structure; MERTK, proto-oncogene tyrosine-protein kinase MER; MGL1, macrophage galactose-type lectin-1; TGM2, transglutaminase 2; MMR (CD206), macrophage mannose receptor; SR, scavenger receptor; YM1/CHI3L3, chitinase-3-like protein-3; information adapted from [39,40,42,50,51,52,53,54,55,56].

**Table 2:** Main effects of maintenance immunosuppressive agents on macrophages.

Immunosuppressive	Mode of Action	Effect on Macrophages	Ref.
rapamycin	inhibition of mTOR (kinase)	impairs macrophage development/infiltration reduces inflammatory cytokine production inhibits polarization to M2-like macrophages promotes development of regulatory macrophages	[97,120,121,122,123,124]
tacrolimus	inhibition of calcineurin (phosphatase)	lowers pro-inflammatory cytokine production reduces expression of M1 surface marker shifts surface markers to M2 phenotype reduces response to LPS reduces NF-κB activation	[125,126,127,128]
cyclosporine A	inhibition of calcineurin (phosphatase)	reduces response to LPS reduces NF-κB activation increases macrophage filtration and fibrosis (compared to tacrolismus)	[129,130,131,132]
mycophenolate mofetil	inhibition of nucleotide synthesis	lowers pro-inflammatory mediators favors M2-like macrophage phenotype increases M2-like surface markers on M1-stimulated macrophages reduces monocyte/endothelial cell interactions	[126,133,134,135]
glucocorticoids	regulation of gene expression	downregulates pro-inflammatory cytokines influences NO production increases macrophage infiltration induces a M2-like phenotype promotes phagocytosis mimics pro-fibrotic phenotype	[136,137,138,139,140,141,142,143]
belatacept	blocking of co-stimulatory molecules CD80/86	enhances phagocyte cell-surface markers reduces migration capacity reduces pro-inflammatory cytokine production induces M1 to M2 macrophage shift	[144,145,146,147,148]

## Data Availability

Not applicable.

## Abbreviations

4-ABH4, 4-amino-BH4; A2R, A2 adenosine receptor; AGMO, alkylglycerol monooxygenase; APC, antigen presenting cell; ARG, arginase; BH4, tetrahydrobiopterin; CBMPs, cell-based medicinal products; CnB1, calcineurin B1; COX, cyclooxygenase; CsA, CTLA-4, cytotoxic T-lymphocyte-associated protein 4; cyclosporine A; CTGF, connective tissue growth factor; DAMPs, damage-associated molecular patterns; DHFR, dihydrofolate reductase; eNOS, endothelial nitric oxide synthase; FAO, fatty acid oxidation; FAS, fatty acid synthesis; FGF-2, fibroblast growth factor 2; GC, glucocorticoids; GCH1, GTP-cyclohydrolase 1; GR, glucocorticoid receptor; GTP, guanosine triphosphate; HDL, high-density lipoprotein; HIF1α, hypoxia-inducible factor 1α; ICAM-1, intracellular adhesion molecule 1; ICs, immune complexes; IDO, indoleamine-2,3-dioxygenase; IFN, interferon; IGF, insulin-like growth factor; IL, interleukin; iNOS, inducible nitric oxide synthase; IP10, IFNγ-inducible protein 10; IRF, interferon-regulatory factor; IRI, ischemia reperfusion injury; KIM-1, kidney injury molecule; LDL, low-density lipoprotein; L-NIL, N-(iminoethyl)-L-lysine; LPS, lipopolysaccharides; MCP-1 (CCL2), monocyte chemoattractant protein 1; M-CSF, macrophage colony-stimulating factor; MIP, macrophage inflammatory protein; MMF, mycophenolate mofetil; MPA, mycophenolic acid; Mregs, regulatory macrophages; MSC, mesenchymal stem cells; mTOR, mammalian target of rapamycin; NF-κB, nuclear factor kappa B; NO, nitric oxide; nNOS, neuronal nitric oxide synthase; NRF2, nuclear factor erythroid 2-related factor 2; OXPHOS, oxidative phosphorylation; PAH, phenylalanine hydroxylase; PBMC, peripheral blood mononuclear cell; PDGF, platelet-derived growth factor; PEAP, progestagen-associated endometrial protein; PIR-A, paired immunoglobulin-like receptors-A; PPAR, peroxisome proliferator-activated receptor; PPP, pentose phosphate pathway; PRR, pattern recognition receptors; PTPS, 6-pyruvoyl tetrahydropterin synthase; PUFA, polyunsaturated fatty acid; rAIM, recombinant apoptosis inhibitor of macrophages; RNS, reactive nitrogen species; ROS, reactive oxygen species; SCID, severe combined immunodeficiency; sFgl2, soluble fibrinogen-like protein 2; SOCS, suppressor of cytokine signaling; SR, sepiapterin reductase; STAT, signal transducer and activator of transcription; TAMs, tumor-associated macrophages; TEC, tubular epithelial cell; TGF, transforming growth factor; TH, tyrosine hydroxylase; TIGIT, T-cell immunoreceptor with Ig and ITIM domains; TLR, toll-like receptors; TNF, tumor necrosis factor; TPH, tryptophan hydroxylase; TRM, tissue-resident macrophages; Treg, regulatory T-cells; VEGF, vascular endothelial growth factor.
